# Expression and Regulation of the *Fkbp5* Gene in the Adult Mouse Brain

**DOI:** 10.1371/journal.pone.0016883

**Published:** 2011-02-09

**Authors:** Sebastian H. Scharf, Claudia Liebl, Elisabeth B. Binder, Mathias V. Schmidt, Marianne B. Müller

**Affiliations:** 1 Research Group Molecular Stress Physiology, Max Planck Institute of Psychiatry, Munich, Germany; 2 Research Group Molecular Genetics of Affective Disorders, Max Planck Institute of Psychiatry, Munich, Germany; 3 Research Group Neurobiology of Stress, Max Planck Institute of Psychiatry, Munich, Germany; University of Birmingham, United Kingdom

## Abstract

**Background:**

Chronic stress has been found to be a major risk factor for various human pathologies. Stress activates the hypothalamic-pituitary-adrenal (HPA) axis, which is tightly regulated via, among others, the glucocorticoid receptor (GR). The activity of the GR is modulated by a variety of proteins, including the co-chaperone FK506 binding protein 51 (FKBP5). Although FKBP5 has been associated with risk for affective disorders and has been implicated in GR sensitivity, previous studies focused mainly on peripheral blood, while information about basal distribution and induction in the central nervous system are sparse.

**Methodology/Principal Findings:**

In the present study, we describe the basal expression pattern of *Fkbp5* mRNA in the brain of adult male mice and show the induction of *Fkbp5* mRNA via dexamethasone treatment or different stress paradigms. We could show that *Fkbp5* is often, but not exclusively, expressed in regions also known for GR expression, for example the hippocampus. Furthermore, we were able to induce *Fkbp5* expression via dexamethasone in the CA1 and DG subregions of the hippocampus, the paraventricular nucleus (PVN) and the central amygdala (CeA). Increase of *Fkbp5* mRNA was also found after restrained stress and 24 hours of food deprivation in the PVN and the CeA, while in the hippocampus only food deprivation caused an increase in *Fkbp5* mRNA.

**Conclusions/Significance:**

Interestingly, regions with a low basal expression showed higher increase in *Fkbp5* mRNA following induction than regions with high basal expression, supporting the hypothesis that GR sensitivity is, at least partly, mediated via *Fkbp5*. In addition, this also supports the use of *Fkbp5* gene expression as a marker for GR sensitivity. In summary, we were able to give an overview of the basal expression of fkbp5 mRNA as well as to extend the findings of induction of *Fkbp5* and its regulatory influence on GR sensitivity from peripheral blood to the brain.

## Introduction

Acute and chronic stress appear on the list of risk factors for a steadily growing number of diseases. These illnesses range from cardiovascular diseases [1] over metabolic disturbances [2] to affective disorders [3], [4], [5]. However, not every individual experiencing a stressful episode will develop a disease, indicating that the complex interplay of multiple factors is decisive. Genetic predispositions, including polymorphisms in the gene encoding the co-chaperone FK506 binding protein 51 (FKBP5), have been implicated as one of these factors by various studies and specifically gene x environment interactions are able to account for increases in the risk to develop a stress-associated disorder [6], [7].

One of the main regulators of the mammalian stress response is the hypothalamic-pituitary-adrenal (HPA) axis. The main effector of this axis, the glucocorticoid hormone corticosterone (cortisol in humans), binds to two different receptors: the high-affinity mineralocorticoid receptor (MR) and the low affinity glucocorticoid receptor (GR). While the MR is already occupied under basal corticosterone levels, the GR needs higher levels to be activated, making the GR more prominent in the negative feedback loop [3]. The GR itself forms a heterocomplex with several other proteins, including the chaperone heat-shock protein 90 (hsp90) as well as the co-chaperone FKBP5, which regulates the folding and trafficking of the GR [8], [9]. In squirrel monkeys, FKBP5 was found to reduce GR binding [10] and it could also be shown that FKBP5 delays translocation of the GR to the nucleus in various mammalian cell lines [11]. As FKBP5 expression can also be induced via GR activation, the system forms an ultra-short feedback loop to regulate GR sensitivity and has been described as an important marker for GR sensitivity and bioavailability [12].

Disruptions of this feedback or HPA-axis deficiencies in general have been associated with various affective disorders, stimulating discussion about an influence of genetic variants of HPA axis related genes, including *Fkbp5*, on these effects [3]. In addition, early trauma has been shown to exert long-lasting effects on HPA-axis regulation [13], [14], [15]. Genes that influence the latter, including *Fkbp5*, are thus in a central position to mediate long term effects of stress. Functional polymorphisms have been described in the *Fkbp5* gene and these are associated with differences in GR-function [16], [6] as well an impaired negative feedback of the HPA-axis [17]. Intriguingly, the alleles that are associated with a prolonged cortisol response following a psychological stressor are the very same that are associated with an increased risk for psychiatric symptoms, including major depression [18], [19], [20], bipolar disorder [21], suicide attempts [22], [23] and PTSD or related phenotypes [24], [6], [25].

Interestingly, a number of these genetic associations seem to be mediated by gene x environment interactions. Only in the presence of early trauma, these *Fkbp5* risk alleles are associated with mentioned psychiatric outcomes [6], [25], [22]. Dysregulation of FKBP5 function and thereby GR-sensitivity in the aftermath of a traumatic event might therefore be an important mediator of long-term negative effects of trauma exposure.

Taken together, there is strong evidence to suggest an important regulatory function of fkbp5 on modulating stress-related brain functions and mechanisms underlying stress-associated psychiatric disorders in humans. While a number of publications have reported genotype effects of variations in the *Fkbp5* gene, there is a lack of information on the basal distribution as well as the regulation of *Fkbp5* gene expression by stress, especially in the central nervous system. A study with post-mortem brains of HIV victims investigated RNA as well as protein levels of *Fkbp5* in the prefrontal cortex [26] and prenatally stressed rats showed differences in FKBP5 protein levels in the frontal cortex, but not in the hippocampus, which could be returned to baseline via treatment with various antidepressant drugs [27]. Nevertheless, many aspects of fkbp5 and its regulation still remain obscure. In the present study, we therefore investigated the detailed neuroanatomical expression pattern of *Fkbp5* mRNA in the murine brain and the effects of stress as well as exogenous glucocorticoid treatment on its regulation.

## Materials and Methods

### Animals

For all experiments, male C57Bl/6N mice (Charles River Laboratories, Maastricht, the Netherlands) at the age of 12 weeks were used. The mice were held under standard conditions (12L:12D light cycle, lights on at 0600 h, temperature 23±2°C) in Plexiglas cages (45×25×20 cm) and were allowed to acclimate to the room for at least 2 weeks before the beginning of the experiments. Food (Altromin 1324, Altromin GmbH, Germany) and tap water were available *ad libitum*, unless specified otherwise. All experiments were carried out in the animal facilities of the Max Planck Institute of Psychiatry in Munich, Germany. The experiments were carried out in accordance with the European Communities' Council Directive 86/609/EEC under the permit ids 55.2-1-54-2531-79-05 and 55.2-1-54-2531-6-09. All efforts were made to minimize animal suffering during the experiments. The protocols were approved by the committee for the Care and Use of Laboratory animals of the Government of Upper Bavaria, Germany.

### Sampling procedure

In order to obtain whole brains of the animals, mice were sacrificed under isoflurane anaesthesia via decapitation. Directly thereafter, the brain was carefully extracted, snap-frozen in pre-cooled 2-methylbutane and stored at −80°C until further processing. In addition, trunk blood was collected in microcentrifuge tubes, immediately put on ice and centrifuged at 4°C and 8000 rpm for 15 minutes. Plasma was then stored at −20°C until further processing.

### Radioimmunoassay

To determine the concentrations of corticosterone in the plasma of the animals, a radioimmunoassay (RIA) was performed using a commercially available kit (ImmunoChemTM Double Antibody Corticosterone 125I RIA Kit, MP Biomedicals, LLC, Orangeburg, NY) with a detection limit of 7.7 ng/ml following the standard protocol.

### Experiment 1 – Qualitative mapping of basal and stress-induced expression of fkbp5 mRNA

A qualitative mapping of the *Fkbp5* mRNA profile was conducted. In addition, we wanted to get qualitative information about the stress regulation of *Fkbp5* mRNA for a more precise design of the follow-up experiments. Animals were either left undisturbed (CON, n = 2) or subjected to 24 h of food deprivation (FD, n = 2) prior to sacrifice. During the stress, animals were kept in their home cages and had free access to tap water. Two animals per group were used, one brain being cut in the coronal plane, the other in the sagittal plane, to obtain a qualitative overview of *Fkbp5* mRNA expression. Animals were sacrificed directly following the food deprivation period as described before (see sampling procedure).

### Experiment 2 – In-depth analysis of stress-induced changes in fkbp5 mRNA expression

For the investigation of the influence of various stressors on *Fkbp5* gene expression, mice were subjected to different stress paradigms. While control animals (CON, n = 8) were left undisturbed in their home cages, one group of mice was restrained in a plastic tube for 30 minutes (RES, n = 8). During that time, the mice were unable to move or turn around in the tube. Following stress, mice were returned to their home cages. Animals were sacrificed under isoflurane anaesthesia 4 h after cessation of the stressful episode (see Sampling procedure). Another group was deprived of food for 24 h (FD, n = 8). Mice were sacrificed directly at the end of the stress period in the same way as described above (see sampling procedure). Based on the mapping in experiment 1, the hypothalamic paraventricular nucleus (PVN) and the central amygdala (CeA) were selected as inducible regions with low basal expression and the dorsal hippocampus as a region with high basal expression of *Fkbp5* mRNA.

### Experiment 3 – Time course of GR-mediated changes in fkbp5 mRNA expression

To further specify the time course of the gene activation, mice were treated with dexamethasone, a potent, synthetic agonist of the glucocorticoid receptor. Animals were injected sub-cutaneously (s.c.) with a single dose of dexamethasone (Dexa-ratiopharm, 100 mg injection solution, Ratiopharm, Germany) at a dosage of 10 mg/kg body weight (BW) with an injection volume of 150 µl per animal (DEX, n = 48). Vehicle-treated animals (VEH, n = 45) were injected with the same amount of saline. Animals were sacrificed 1 h, 4 h, 8 h or 24 h post injection as described before (see sampling procedure). As in experiment 2, the PVN, the CeA and the hippocampus were investigated.

### In-situ hybridisation

To assess brain mRNA levels of *Fkbp5*, *in-situ* hybridisation (ISH) was performed. Frozen brains were mounted in a cryostat microtome and 18 µm sections were cut with a knife temperature of -14°C and a sample temperature of -16°C. Sections were thaw-mounted on superfrost slides, dried shortly and stored at -80°C. Brains were cut in the coronal and sagittal plane, keeping every fourth slice for experiment 1. For experiments 2 and 3, brains were cut at the level of the PVN as well as the dorsal hippocampus. An intron-spanning riboprobe for *Fkbp5* was designed using Primer3 software, spanning from exon 5 to exon 9 of the murine *Fkbp5* gene, with a total length of 491 base pairs (Forward primer 5′-3′: CTTGGACCACGCTATGGTTT; Reverse primer 5′-3′: GGATTGACTGCCAACACCTT). *In-situ* hybridisation using ^35^S-UTP-labelled ribonucleotide probes was performed as described previously [28]. Briefly, sections were fixed in 4% paraformaldehyde and acetylated in 0.25% acetic anhydride. Afterwards, slides were dehydrated in ascending concentrations of alcohol. On the dried slides, hybridization buffer containing between 1.5 and 2.0×10^6^ counts per minute of ^35^S-UTP -labelled riboprobe was applied with a volume of 100 µl per slide. Brain sections were coverslipped and incubated overnight at 55°C. The next day, sections were rinsed and incubated with RNAse A. Finally, sections were desalted and dehydrated.

Radioactively labelled slides were apposed to Kodak Biomax MR films (Eastman Kodak Co., Rochester, NY) and were developed using an automated developing machine. Films were digitized and relative expression was measured by optical densitometry using the ImageJ software (available at http://rsb.info.nih.gov/ij; developed by Wayne Rasband, National Institutes of Health, Bethesda, MD). For each animal, the mean of the bilateral structures of two sections was calculated if applicable, deducting the background from the value. Background signal was measured in a structure not expressing *Fkbp5* mRNA, which was the *stratum radiatum* in the case of the hippocampus and the amygdala as well as the peri-PVN region in case of the PVN. For the qualitative mapping, signal intensities were grouped (- 0-10; +10-20; ++20-60; +++ >60 mean grey levels). Slices were counterstained with kresyl-violet after ISH for histological assessment.

### Data analysis

For statistical analyses, the commercially available program SPSS 17 was used. All gene expression levels are given in arbitrary units (a.u.). Comparisons of three conditions were assessed by One-way ANOVA followed by Bonferroni post-hoc tests. For the multifactorial datasets, Two-way ANOVA was performed, follow by Bonferroni post-hoc tests in case of 3 or more groups or independent-samples T-tests in case of 2 groups. Values outside the 95% confidence interval were defined as outliers and excluded from the analyses. Significance levels were set at p<0.05 and a trend was recognized at p<0.1. Statistics for the tables with the normalised values are the same as for the absolute values. Figures were created using the SigmaPlot software (SigmaPlot 10.0) with help of Adobe Photoshop CS3 and Adobe Illustrator CS3. For representative images, contrast was modified for better display (all analyses were done on unmodified images). Interaural and bregma coordinates for the supplementary figures were taken from a brain atlas [29].

## Results

### Experiment 1

**Figure 1:**
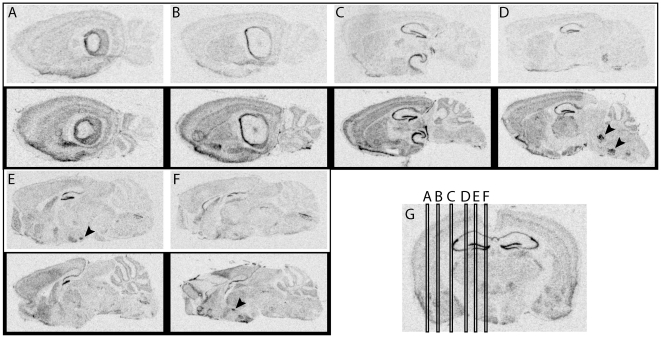
Qualitative overview of fkbp5 mRNA expression in sagittal slices. (**A**)**–**(**F**) Selected slices throughout the brain. (**D**) Black arrowheads point at the motor nuclei of the *nervus trigeminus* and *nervus facialis*. (**E**) Black arrowhead highlights the premammilary nucleus. (**F**) Black arrowhead shows the hypothalamic PVN. (**G**) Coronal overview depicting the level of the selected slices. Autoradiographs with a white frame were from the control condition, autoradiographs with a black frame were from the FD condition.

**Figure 2:**
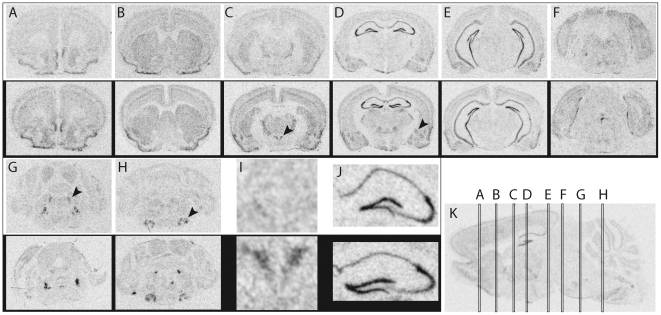
Qualitative overview of fkbp5 mRNA expression in coronal slices. (**A**)**–**(**H**) Selected slices throughout the brain. **(C)** Black arrowhead points at the hypothalamic PVN. (**D**) Black arrowhead shows the central amygdala. (**G**) Black arrowhead highlights the locus coeruleus. (**H**) Black arrowhead focuses on the motor nucleus of the *nervus facialis*. (**I**) Magnification of the hypothalamic PVN from (**C**). (**J**) Magnification of the hippocampus from (**D**). (**K**) Sagittal overview depicting the level of the selected slices. Autoradiographs with a white frame were from the control condition, autoradiographs with a black frame were from the FD condition.

A qualitative overview of *Fkbp5* mRNA expression can be found in Figure 1 for the sagittal plane and in Figure 2 for the coronal plane. A systematic overview can be found in Table 1. The exact definitions of the regions and histological staining can be found in the supplementary Figures S1, S2, S3, S4, S5, S6, S7, S8, S9 and S10. Induction of gene expression via FD was ubiquitous over the brain, but most prominent in regions with the low basal expression like the hypothalamic PVN or the central amygdala.

**Table 1:** Systematic overview of basal *Fkbp5* mRNA expression.

Region	EXP	Region	EXP
*Prosencephalon*		*Prosencephalon (continued)*	
**Telencephalon**		**Diencephalon**	
*Cerebral cortex*		*Thalamus*	
Isocortex		Habenular nuclei	+
Layer 1	-	Reticular nucleus	++
Layer 2	+	Bed nuclei of the stria terminalis	+
Layer 3	-	*Hypothalamus*	
Layer 4	-	Paraventricular nucleus	+
Layer 5	+	Ventral premammilary nucleus	++
Layer 6	-	Anterior hypothalamic area	+
Allocortex		Ventromedial hypothalamic nuclei	++
Septum	++	*Mesencephalon*	
Indusium griseum	++	*Tectum*	
Piriform cortex	++	Inferior colliculus	-
Hippocampus		Superior colliculus	+
Dentate gyrus	+++	*Tegmentum*	
CA1	++	Ventral tegmental nucleus	++
CA2	+++	Cerebral peduncle	-
CA3	++	*Rhombencephalon*	
Subiculum	++	**Metencephalon**	
*Basal ganglia*		Cerebellum	-
Striatum		*Pons*	
Caudate putamen	+	Locus coeruleus	++
Nucleus accumbens		Trigeminal motor nucleus	++
Shell	++	Facial nucleus	++
Core	++	**Myelencephalon**	
Amygdaloid complex		*Medulla oblongata*	
Basolateral nucleus	+	Raphe nuclei	-
Central nucleus	++		
Medial nucleus	++		

- detection similar to background, + low expression, ++ moderate expression, +++ strong expression, Exp  =  expression.

### Experiment 2

**Figure 3:**
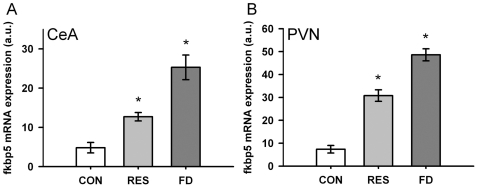
Regulation of fkbp5 mRNA in the central amygdala and the hypothalamic PVN following restrained stress or food deprivation. (**A**) In the central amygdala, fkbp5 mRNA was upregulated 4 h after restrained stress and even more pronounced after 24 h of food deprivation. (**B**) In the hypothalamic PVN, a similar regulation was found. CON  =  control, RES  =  restrained stress, FD  =  food deprivation, CeA  =  central amygdala, PVN  =  paraventricular nucleus. Data are given in mean ± SEM. * different to control p<0.05.

**Figure 4:**
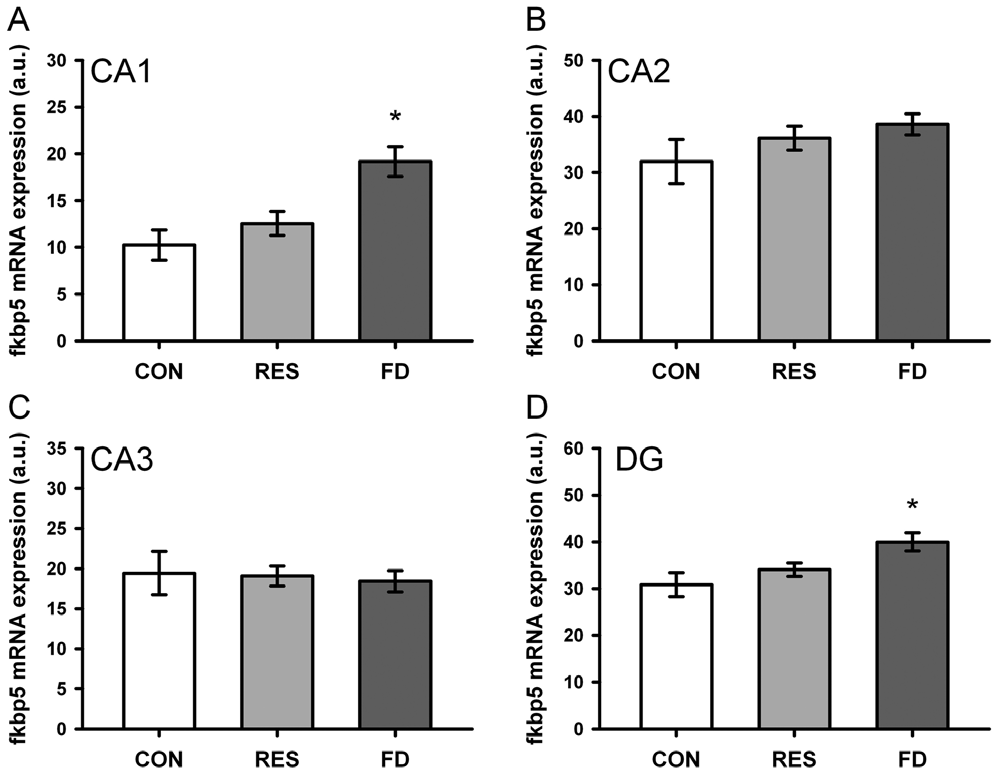
Regulation of fkbp5 mRNA in the dorsal hippocampus following restrained stress or food deprivation. Expression in the (**A**) CA1, (**B**) CA2, (**C**) CA3 and (**D**) DG subregions of the hippocampus. Upregulation of fkbp5 mRNA can be seen in the CA1 region as well as in the DG after 24 h of food deprivation. CON  =  control, RES  =  restrained stress, FD  =  food deprivation. Data are given in mean ± SEM. * different to control p<0.05.

*Fkbp5* gene expression was analysed in unstressed control animals, 4 h after restrained stress and at the end of a 24 h food deprivation period. Corticosterone levels can be found in Table 2 and showed a condition effect (One-Way ANOVA; F_2,21_ = 26.794, p<0.01) with significantly increased levels in FD, but not in restrained animals. Gene expression was investigated in the central amygdala, the hypothalamic PVN and the hippocampus (see Figures 3A, 3B and 4, respectively). In case of the central amygdala, One-way ANOVA showed a significant effect between the conditions (One-Way ANOVA; F_2,20_ = 23.647, p<0.01) and post-hoc testing showed increased level of *Fkbp5* gene expression in restrained animals compared to controls as well as increased levels for food deprived animals compared to both controls and restrained animals. The same effect was found in the PVN (One-Way ANOVA; F_2,22_ = 81.214, p<0.01). In addition, the data showed different regulation of *Fkbp5* mRNA after different stressors in distinct sub-regions of the hippocampus. *Fkbp5* mRNA was up-regulated in the CA1 and the DG sub-regions following food deprivation (One-way ANOVA; CA1: F_2,22_ = 9.498, p<0.01; DG: F_2,22_ = 5.437, p<0.05), but not following restrained stress (see Figure 4). Neither restraint nor food deprivation had any influence on *Fkbp5* mRNA levels in the CA2 and CA3 regions. For a better comparison between regions with low (CeA and PVN) and high (hippocampus) basal *Fkbp5* mRNA expression, relative increases are displayed in Table 3.

**Table 2:** Corticosterone levels in experiment 2.

Condition	Corticosterone [ng/ml]
Control	16.6±2.9
Restrained	32.0±6.9
Food deprivation	506.5±86.4* †

Data are given in mean ± SEM.

*different to control p<0.05,

† different to restrained p<0.05.

**Table 3:** Increase in fkbp5 mRNA following different stress paradigms.

Region	Condition		
	Control	Restrained	Food deprivation
***low basal fkbp5 mRNA expression***			
CeA	100±28	264±22	524±65
PVN	100±22	419±34	661±36
***high basal fkbp5 mRNA expression***			
*Hippocampus*			
CA1	100±16	122±13	187±16
CA2	100±12	113±7	120±6
CA3	100±14	98±6	95±7
G	100±8	110±5	114±6

Data are given in percentage of the mean ± SEM. Values are normalised to control animals.

### Experiment 3

**Figure 5:**
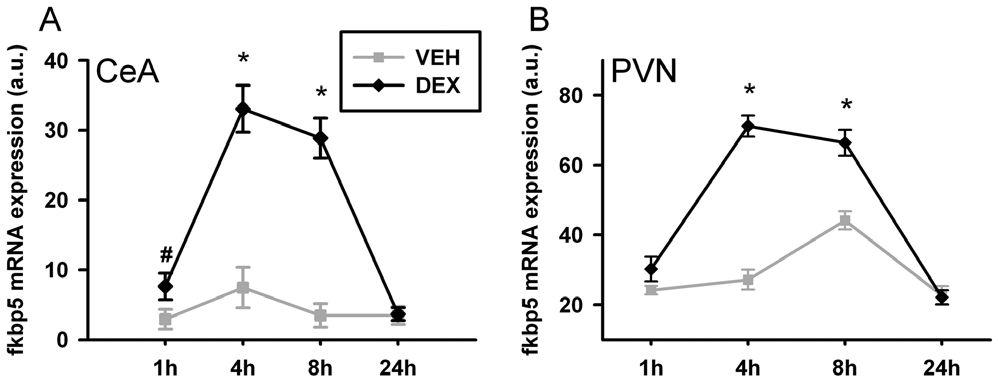
Temporal regulation of fkbp5 mRNA in the central amygdala and the hypothalamic PVN following dexamethasone injection. (**A**) Fkbp5 mRNA was significantly upregulated 4 h and 8 h after dexamethasone treatment in the central amygdala, but returned to baseline after 24 h. Upregulation can already be detected 1 h after injection as a trend. (**B**) The same holds true for the hypothalamic PVN. In addition, an effect of the vehicle injection can be seen with an increase after 8 h. VEH  =  injected with vehicle, DEX  =  injected with dexamethasone, CeA  =  central amygdala, PVN  =  paraventricular nucleus. Data are given in mean ± SEM. * different to vehicle p<0.05, # different to vehicle p<0.1.

**Figure 6:**
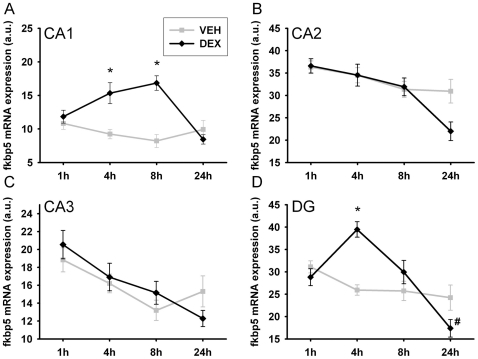
Temporal regulation of fkbp5 mRNA in the hippocampus following dexamethasone injection. (**A**) Modulation of fkbp5 mRNA expression in the CA1 subfield of the hippocampus. An up-regulation was found 4 and 8 h after dexamethasone treatment. (**B**) No significant overall effect was found for the CA2 region. (**C**) In the CA3 region, dexamethasone treatment had no effect. (**D**) Fkbp5 shows a differential regulation in the DG with a significant increase 4 h after injection. VEH  =  injected with vehicle, DEX  =  injected with dexamethasone. Data are given in mean ± SEM. * different to vehicle p<0.05, # different to vehicle p<0.1.

The temporal regulation of *Fkbp5* mRNA expression was investigated by injection of dexamethasone. In the central amygdala, *Fkbp5* gene expression was elevated significantly after the injection (Two-way ANOVA; treatment: F_1,45_ = 84.407, p<0.01; timepoint: F_3,45_ = 28.673, p<0.01; treatment*timepoint interaction: F_3,45_ = 19.361, p<0.01), with significantly increased levels 4 h and 8 h after injection and a trend after 1 h (T-test; 1 h: T_10_ = 1.971, p = 0.077; 4 h: T_9_ = 5.812, p<0.01; 8 h: T_9_ = 7.960, p<0.01). Gene expression returned to basal levels 24 h post injection (see Figure 5A). A similar effect was demonstrated for the PVN (Two-way ANOVA; treatment: F_1,46_ = 78.072, p<0.01; timepoint: F_3,46_ = 62.030, p<0.01; treatment*timepoint interaction: F_3,46_ = 25.188, p<0.01), also with significant elevations in *Fkbp5* mRNA levels 4 h and 8 h following injection (T-test; 4 h: T_10_ = 10.597, p<0.01; 8 h: T_8_ = 4.364, p<0.01). In contrast to the central amygdala, an effect of vehicle injection was seen 8 h after injection in the PVN (see Figure 5B). In concordance with the second experiment, the heterogeneous hippocampal region showed different patterns of regulation in the distinct sub-regions (see Figure 6). ANOVA revealed differences in gene expression in the CA1 region (Two-way ANOVA; condition: F_1,93_ = 22.817, p<0.01; timepoint: F_3,93_ = 3.900, p<0.05; condition*timepoint interaction: F_3,93_ = 9.383, p<0.01) with elevated *Fkbp5* mRNA levels at the 4 h and 8 h time points (T-test; 4 h: T_22_ = 3.540, p<0.01; 8 h: T_22_ = 5.935, p<0.01). For the CA2 and CA3 regions, a significant effect of time point was found, but no effect of condition (Two-way ANOVA; CA2: condition: n.s.; timepoint: F_3,94_ = 9.396, p<0.01; condition*timepoint interaction: F_3,94_ = 2.620, p = 0.056; CA3: condition: n.s.; timepoint: F_3,93_ = 8.311, p<0.01; condition*timepoint interaction: n.s.). Finally, investigation of the DG revealed differences over time based upon dexamethasone treatment (Two-way ANOVA; condition: n.s.; timepoint: F_3,93_ = 12.347, p<0.01; condition*timepoint interaction: F_3,93_ = 9.785, p<0.01). Post-hoc analysis specified the temporal alterations in *Fkbp5* mRNA levels with a significant increase 4 h after injection (T-test; 4 h: T_22_ = 6.512, p<0.01) followed by a trend for decrease at 24 h (T-test; 24 h: T_22_ = 2.064, p = 0.053). Relative increases are displayed in Table 4.

**Table 4:** Increase in fkbp5 mRNA following dexamethasone injection.

Region	Time after DEX injection			
	1 h	4 h	8 h	24 h
***low basal fkbp5 mRNA expression***				
CeA	260±65	443±45	833±83	105±27
PVN	125±14	262±11	150±8	98±9
***high basal fkbp5 mRNA expression***				
*Hippocampus*				
CA1	109±9	166±17	205±13	85±7
CA2	101±4	100±7	102±6	71±7
CA3	109±8	104±10	115±10	80±6
DG	93±6	152±7	116±10	73±9

Data are given in percentage of the mean ± SEM. Values are normalised to the respective vehicle-treated animals.

## Discussion

In our experiments, we demonstrated that there is an ubiquitous basal expression of *Fkbp5* mRNA throughout the adult mouse brain with higher expression levels in specific brain areas. In addition, we were able to induce elevated gene expression with various stressors as well as dexamethasone treatment. Interestingly, temporal modulation varied between different brain regions, even within the hippocampus.

Under basal conditions, *Fkbp5* mRNA was most strongly expressed in regions also known to express the GR [30], [31], such as the hippocampal formation [32], [33], the premammillary nucleus [34] or the motor nuclei of the *nervus trigeminus* and *nervus facialis* [35]. Intriguingly, in the hypothalamic PVN, a central part of the HPA axis itself and also a mediator of stress response feedback, basal expression of *Fkbp5* mRNA was very low. Given the fact that the FKBP5 protein is an important part of the chaperone complex regulating GR function [9], expression patterns similar to GR expression reinforce the importance of FKBP5 in modulating GR function.

We have also demonstrated that *Fkbp5* mRNA expression was inducible via treatment with dexamethasone, a synthetic glucocorticoid with a high affinity for the GR, and different stress paradigms. A number of studies have shown that *Fkbp5* mRNA expression can be induced via glucocorticoid treatment in cells *ex vivo* or in culture in a concentration dependent manner [12], [36], [37] and a strong positive correlation between *Fkbp5* mRNA and cortisol has been reported in whole blood [16]. A recent study has also shown that chronic treatment with corticosterone increased *Fkbp5* mRNA expression in the hypothalamus and the hippocampus as well as in peripheral blood [38]. Thus, we were able to replicate and extend these findings *in vivo* to the central nervous system, suggesting that the proposed intracellular ultra-short feedback loop is also relevant for this tissue. In addition, we could show that not only direct stimulation of the GR by dexamethasone, but also stress-induced increases in corticosterone are able to strongly induce *Fkbp5* expression in the CNS. This further supports the physiological relevance of *Fkbp5* regulation during GR activation.

In the present study, we used two different stress paradigms, restrained stress, which is believed to be a moderate stressor, as well as food deprivation (FD), representing a strong and long-lasting stressor. This is also reflected in higher corticosterone increases in the FD condition. It should be noted here that the presented corticosterone levels in the restrained condition were take 4 hours after cessation of the stressor and thereby have likely already normalised due to negative feedback. Moreover, food deprivation is not only quantitatively different, but also qualitatively. As the animals are deprived of food for 24 h, this stressor also includes a metabolic component. In the hypothalamic PVN as well as in the CeA, we were able to show a dose-dependent increase in *Fkbp5* mRNA, with higher levels in the FD condition and intermediate levels following restrained stress.

Not only the severity of the stressor - and thus corticosterone levels - influence the extent of *Fkbp5* mRNA induction, but also baseline expression of *Fkbp5*. Regions with low *Fkbp5* mRNA baseline expression, namely the PVN and the CeA, showed a strong increase in *Fkbp5* mRNA expression levels, which could already be induced via the milder restraint stress. In contrast, increase in Fkbp5 mRNA was not so prominent in the hippocampus, a region with strong basal *Fkbp5* expression. In the latter case, higher corticosterone levels are required, as restraint stress proved to be ineffective in inducing mRNA expression. Although we cannot complete rule out the possibility of a simple ceiling effect in the hippocampus, we think it is highly unlikely. The strongest increase in mRNA in the hippocampus is 187% (see Table 3) and we do not think this is already saturation, as in the PVN and the CeA increases up to 661% are possible and data from cell lines (lymphoblast IM9) show increases of up to 1000% [12]. This supports the hypothesis that *Fkbp5* is critically involved in regulating GR sensitivity and higher baseline levels of *Fkbp5* mRNA result in a higher resistance of the GR to glucocorticoid hormones, as GR mediated induction of *Fkbp5* mRNA was proposed as a readout of GR sensitivity [39], [40]. Therefore, local baseline *Fkbp5* mRNA levels can act as a marker of GR sensitivity in that specific brain region.

Regarding the temporal dynamics of the gene expression changes, we found that mRNA expression was up-regulated in the CeA, the PVN and the hippocampal CA1 region 4 hours and 8 hours following dexamethasone injection and returned to baseline after 24 hours. As described for different stress paradigms, also the increase of *Fkbp5* mRNA following exogenous glucocorticoid injection was stronger in low baseline *Fkbp5* regions like the PVN and the CeA and weaker in the hippocampus, with high basal *Fkbp5* mRNA expression. The return to baseline expression levels after 24 hours is in contrast to the findings of Vermeer et al. showing an increase in expression 4 hours and 8 hours after dexamethasone treatment, but also describing elevated levels of *Fkbp5* mRNA 24 hours after treatment. This displays the differences to cell culture experiments, most likely in kinetics and dynamics, and highlights the importance of *in vivo* studies. Besides the latter possibilitites, extracellular influence might also account for these differences. Interestingly, we found that normalisation is heterogeneous even within the subregions of the hippocampus, as the temporal pattern in the DG subregion showed normalisation after 8 hours and a substantial overshoot 24 hours after the treatment. Moreover, vehicle-treated animals also show slight temporal variations in *Fkbp5* gene expression. This might indicate an influence of the circadian rhythm on *Fkbp5* mRNA expression, but further studies are needed for more specific statements.

All these pieces of evidence might have important implications on our understanding of the effects of chronic stress exposure as well as human pathology. Chronic stress in general is accompanied by, among other things, chronically elevated corticosteroid levels. Although some counter-regulatory mechanisms, like receptor down-regulation, are already elucidated [28], the complex interactions of different components of the feedback machinery are still poorly understood. *Fkbp5* could likely be an important factor. As *Fkbp5* mRNA is induced under stressful conditions in specific brain areas and high levels of *Fkbp5* mRNA lead to decreased GR sensitivity, one could hypothesise that chronic stress could induce chronically elevated *Fkbp5* levels, which in turn would decrease GR sensitivity in a long-lasting fashion, potentially causing elevated corticosteroid levels. This would very likely then be a synergistic effect together with previously reported effects.

By now, many links have been found between stress and human diseases. Not only is chronic stress a major risk factor for a multitude of human pathologies [41], [42], [43], inlcuding affective disorders like depression, but also some aspects of illnesses show an overlap with the effects of chronic stress exposure. In the case of depression, about 50% of all patients suffer from elevated cortisol levels, possibly caused by a hyperactive HPA axis [44]. In addition, maladaptions visible in the Dex/CRH test were able to predict relapse and response rates of patients [45], [46]. Human genetic studies have shown that single mutations in the *Fkbp5* gene can have an influence on risk for depression, number of depressive episodes and response to antidepressant treatment (reviewed in Binder et al., 2009), clearly stressing the importance of *Fkbp5* itself in these disorders and the need for further investigation, especially in the form of *in vivo* mammalian models.

Recapitulatory, we were able to describe the basal expression pattern of *Fkbp5* mRNA expression pattern in the murine brain, which is often, but not always, expressed in GR-rich areas. In addition, we demonstrated that *Fkbp5* expression can be induced via two stress paradigms as well as dexamethasone injection in limbic brain structures like the central amygdala and the hippocampus as well as the hypothalamic PVN. Furthermore, we have shown that GR-mediated increases in *Fkbp5* mRNA expression levels were dependent on basal *Fkbp5* gene expression, with higher levels of basal *Fkbp5* mRNA associating with an attenuated response - pointing at a strong role of *Fkbp5* in GR sensitivity.

## Supporting Information

Figure S1 **Measured regions for the mapping.** Coordinates: Interaural 4.98 mm, Bregma 1.18 mm. IG indusium griseum, S septum, CP caudate putamen, Pir piriform cortex (layer 2), NAcC core of the nucleus accumbens, NAcS shell of the nucleus accumbens.
(TIF)

Figure S2 **Measured regions for the mapping.** Coordinates: Interaural 3.58 mm, Bregma -0.22 mm. BNST bed nuclei of the stria terminalis.
(TIF)

Figure S3 **Measured regions for the mapping.** Coordinates: Interaural 2.86 mm, Bregma -0.94 mm. PVN paraventricular nucleus of the hypothalamus, AHA anterior hypothalamic area.
(TIF)

Figure S4 **Measured regions for the mapping.** Coordinates: Interaural 2.74 mm, Bregma -1.06 mm. Hb habenular nucleus, Rt reticular nucleus.
(TIF)

Figure S5 **Measured regions for the mapping.** Coordinates: Interaural 1.86 mm, Bregma -1.94 mm. DG dentate gyrus, VmHN ventromedial hypothalamic nucleus, MA medial amygdala, CeA central amygdala, BLA basolateral amygdala.
(TIF)

Figure S6 **Measured regions for the mapping.** Coordinates: Interaural 1.34 mm, Bregma -2.46 mm. vPmN ventral premammilary nucleus.
(TIF)

Figure S7 **Measured regions for the mapping.** Coordinates: Interaural -0.36 mm, Bregma -4.16 mm. SC superior colliculus, DR dorsal raphe nucleus, VTN ventral tegmental nucleus.
(TIF)

Figure S8 **Measured regions for the mapping.** Coordinates: Interaural -1.54 mm, Bregma -5.34 mm. LC locus coeruleus, TMN trigeminal motor nucleus, IC inferior colliculus.
(TIF)

Figure S9 **Measured regions for the mapping.** Coordinates: Interaural -2.44 mm, Bregma -6.24 mm. FN facial nucleus.
(TIF)

Figure S10 **Identification of the cortex layers.** Coordinates: Interaural 0.26 mm, Bregma 4.06 mm. Only the cortex is shown. I Layer 1, II Layer 2, III Layer 3, IV Layer 4, V Layer 5, VI Layer 6.
(TIF)
