# Supplementary figures and images for: Expression and Regulation of the Fkbp5 Gene in the Adult Mouse Brain
Source: PLoS One. 2011 Feb 9;6(2):e16883. doi: 10.1371/journal.pone.0016883 (PMC3036725; doi:10.1371/journal.pone.0016883)

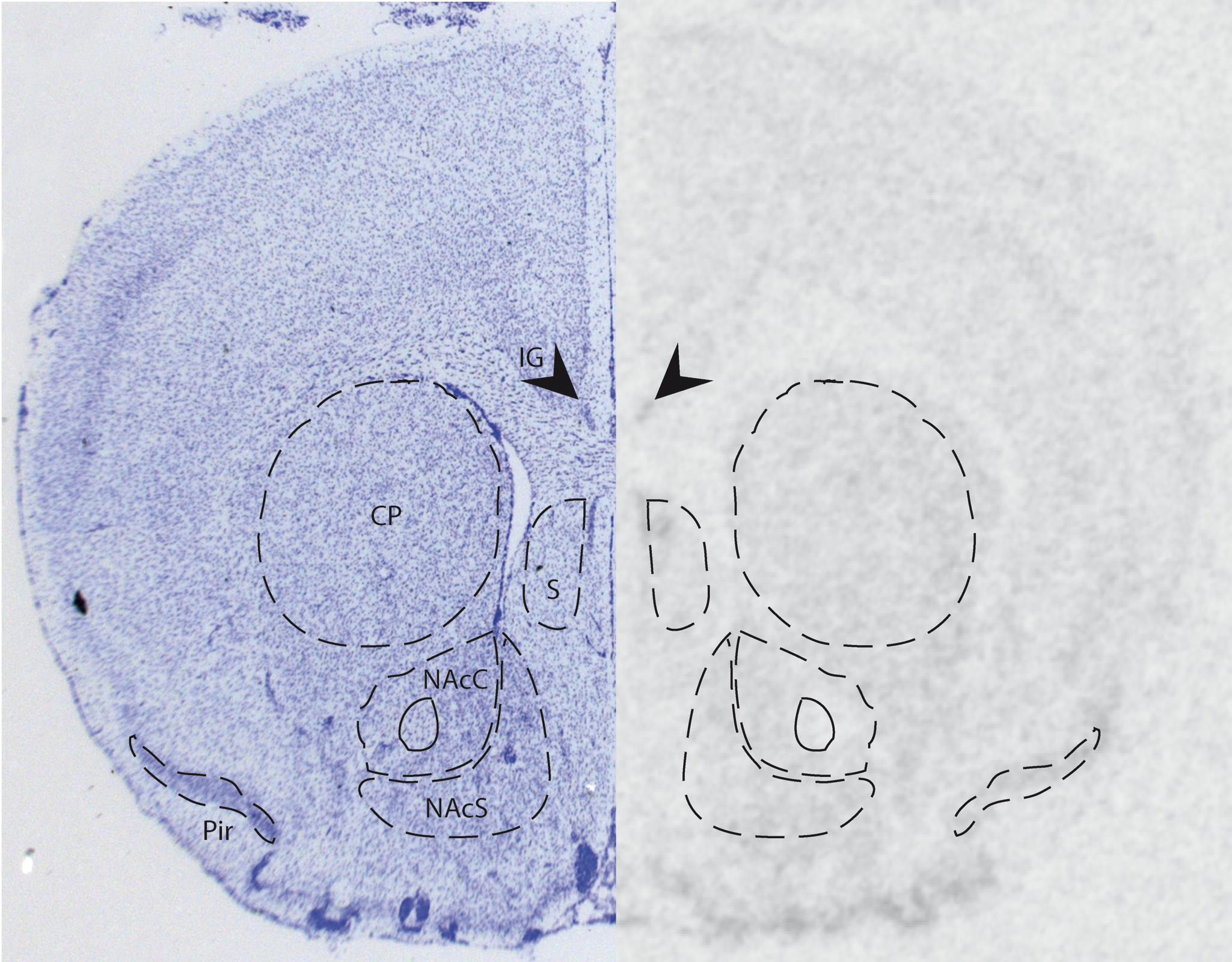

Supplement: Figure S1 — Measured regions for the mapping. Coordinates: Interaural 4.98 mm, Bregma 1.18 mm. IG indusium griseum, S septum, CP caudate putamen, Pir piriform cortex (layer 2), NAcC core of the nucleus accumbens, NAcS shell of the nucleus accumbens. (TIF) [file pone.0016883.s001.tif]

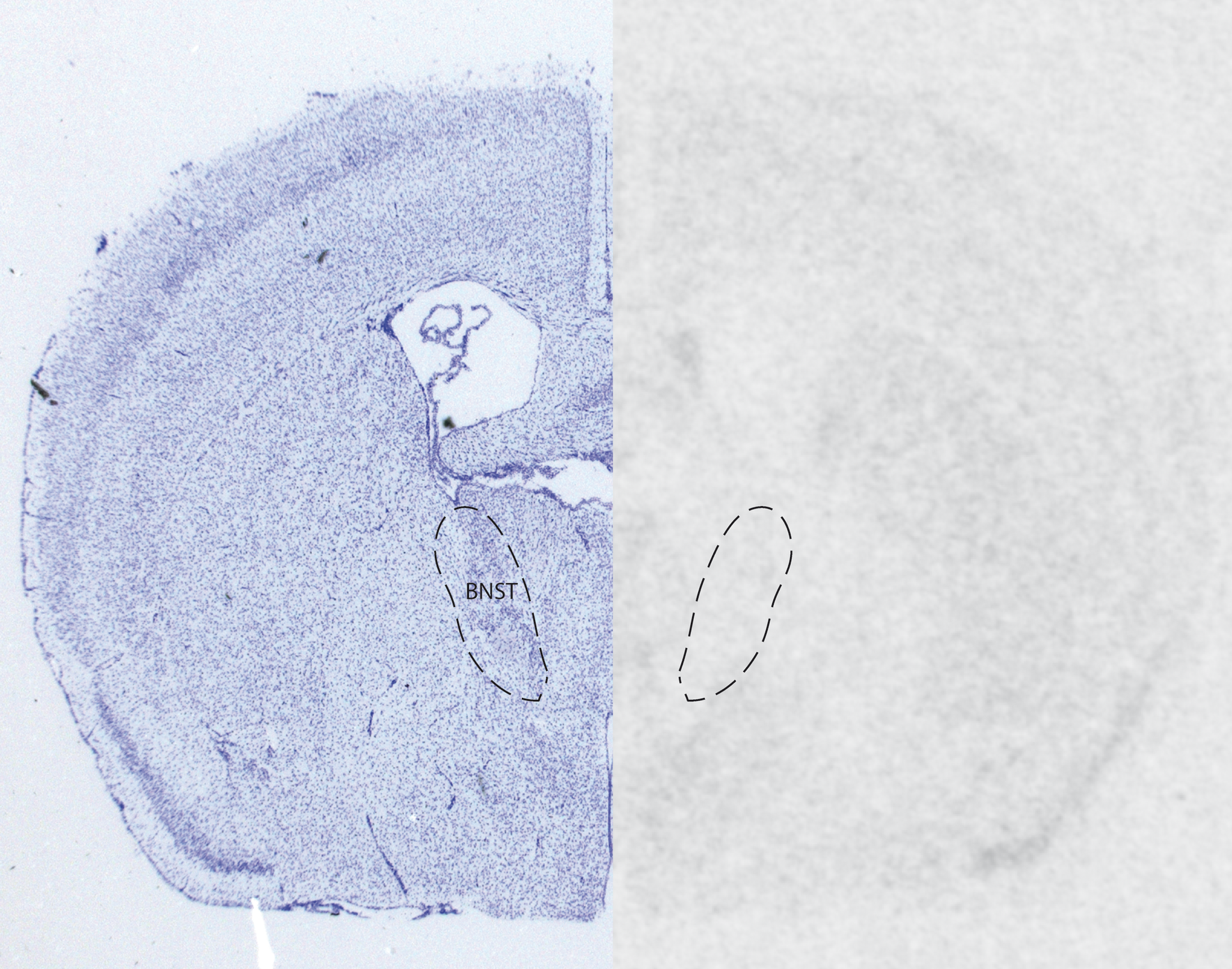

Supplement: Figure S2 — Measured regions for the mapping. Coordinates: Interaural 3.58 mm, Bregma -0.22 mm. BNST bed nuclei of the stria terminalis. (TIF) [file pone.0016883.s002.tif]

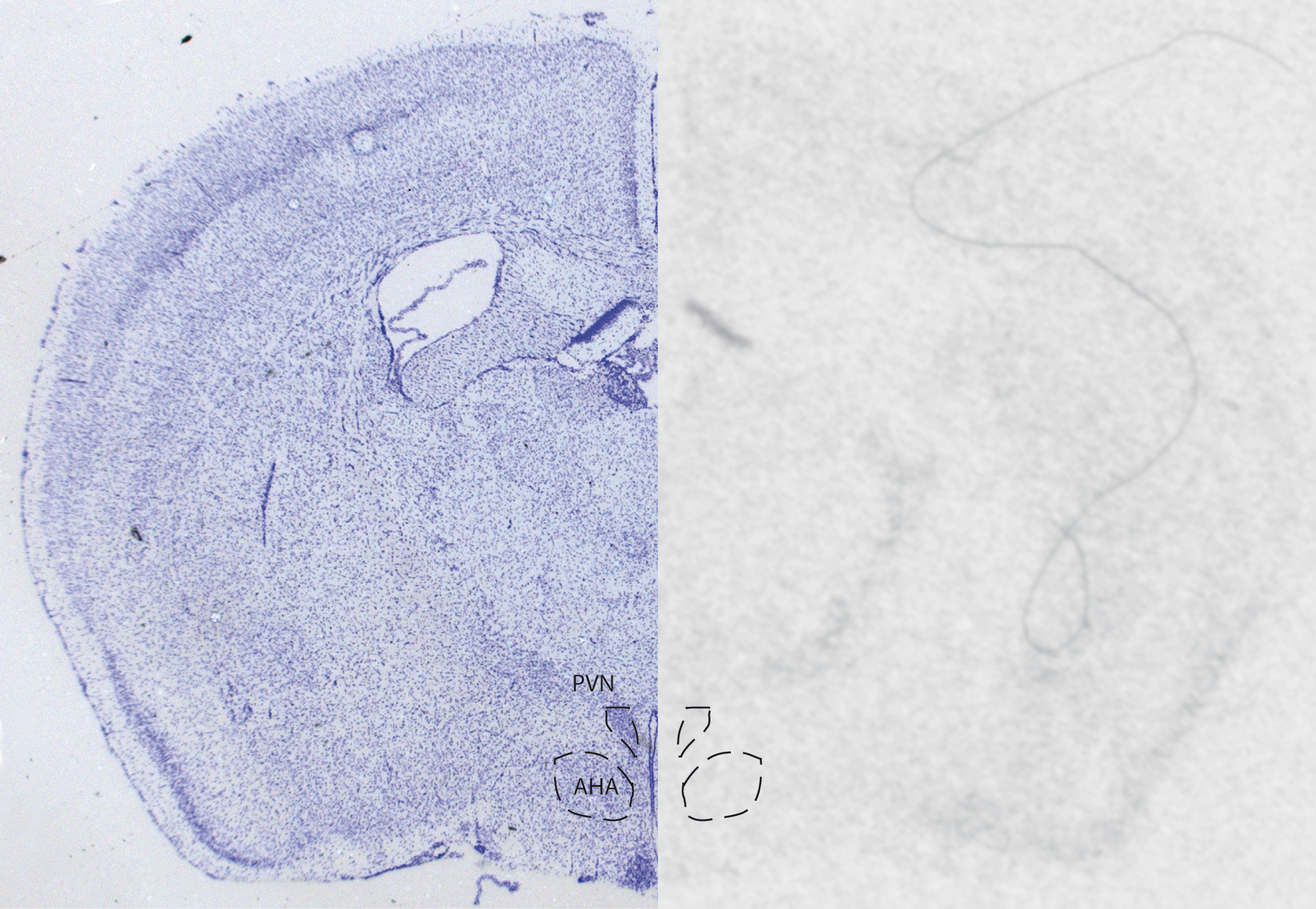

Supplement: Figure S3 — Measured regions for the mapping. Coordinates: Interaural 2.86 mm, Bregma -0.94 mm. PVN paraventricular nucleus of the hypothalamus, AHA anterior hypothalamic area. (TIF) [file pone.0016883.s003.tif]

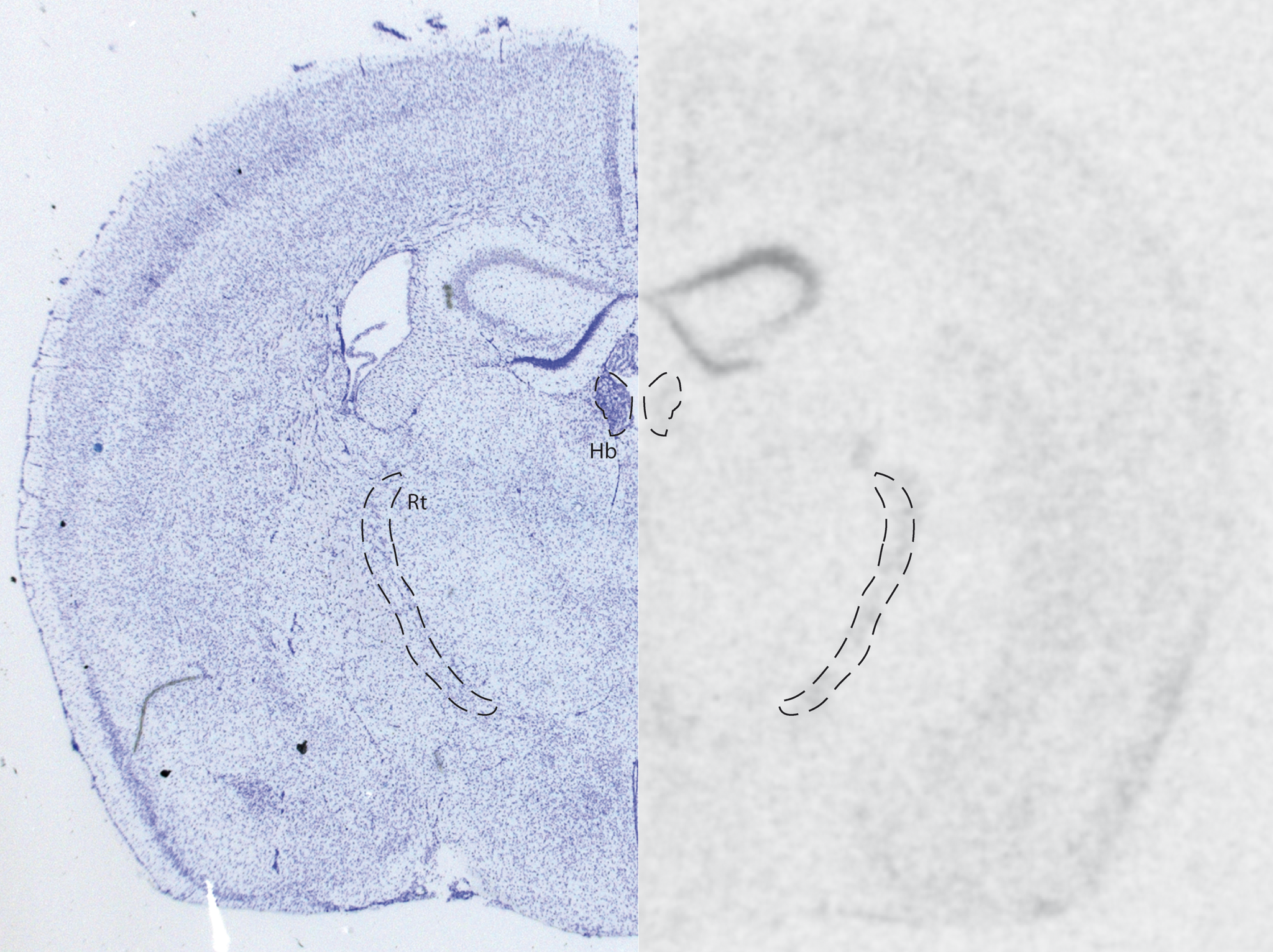

Supplement: Figure S4 — Measured regions for the mapping. Coordinates: Interaural 2.74 mm, Bregma -1.06 mm. Hb habenular nucleus, Rt reticular nucleus. (TIF) [file pone.0016883.s004.tif]

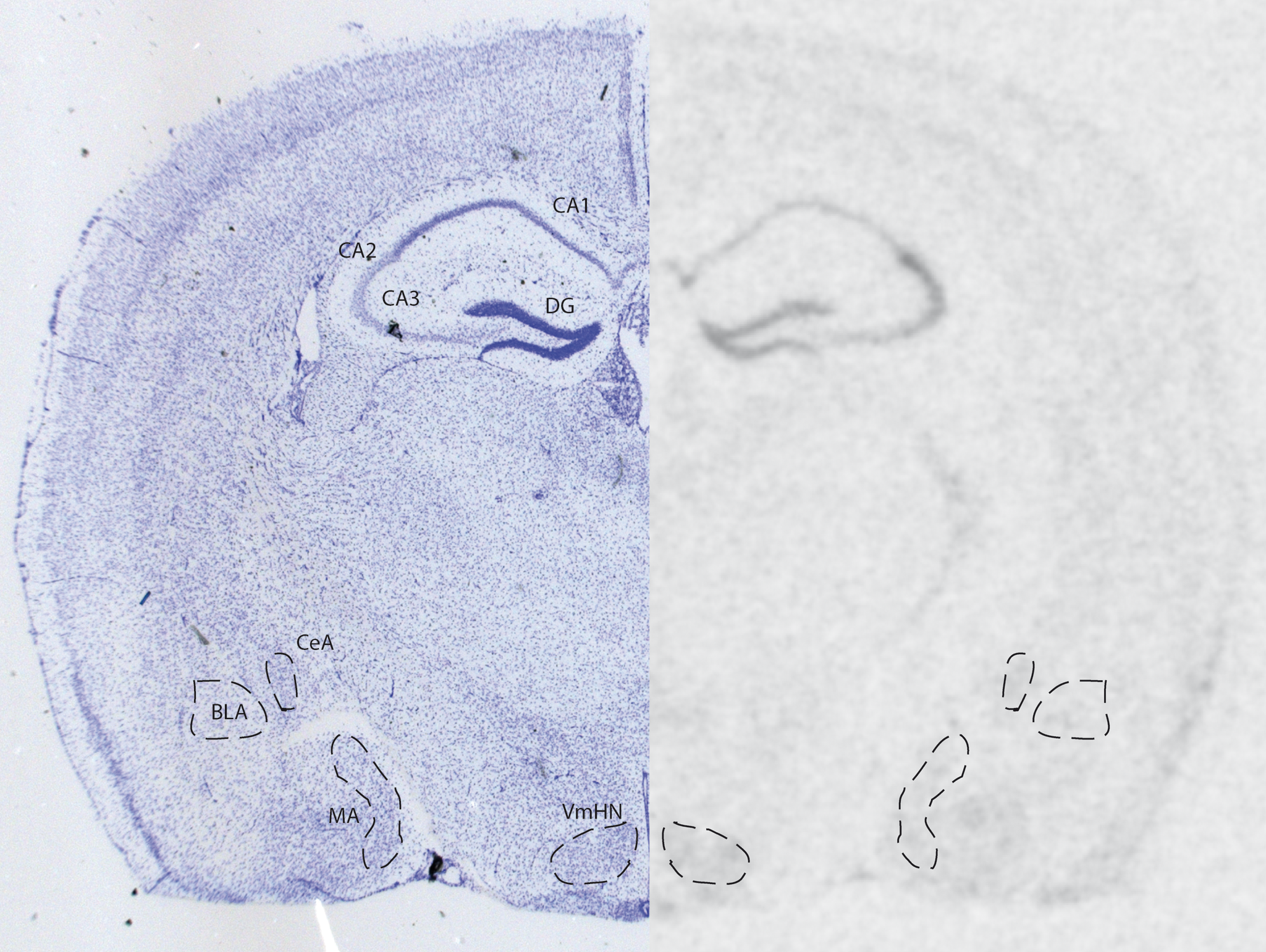

Supplement: Figure S5 — Measured regions for the mapping. Coordinates: Interaural 1.86 mm, Bregma -1.94 mm. DG dentate gyrus, VmHN ventromedial hypothalamic nucleus, MA medial amygdala, CeA central amygdala, BLA basolateral amygdala. (TIF) [file pone.0016883.s005.tif]

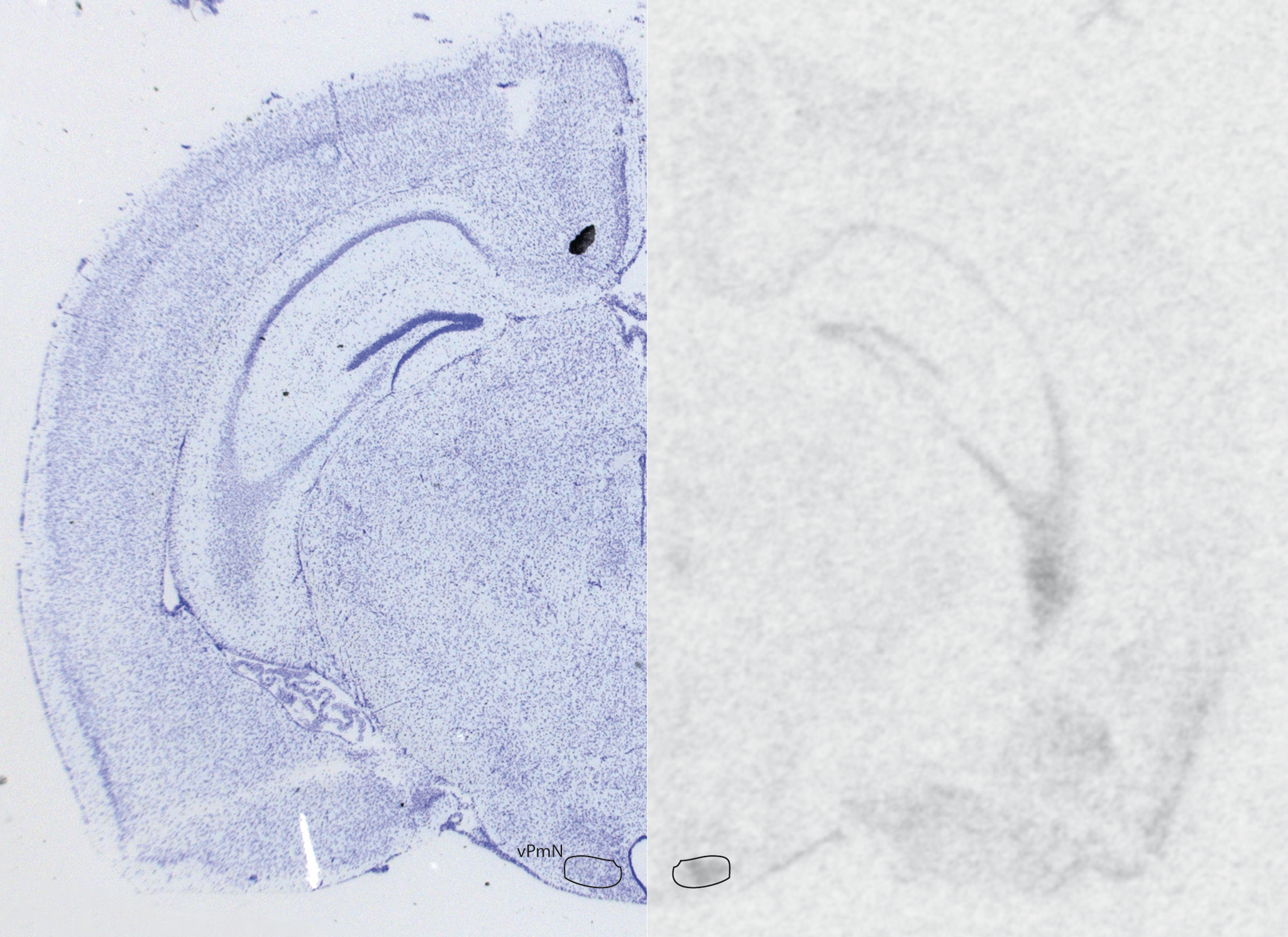

Supplement: Figure S6 — Measured regions for the mapping. Coordinates: Interaural 1.34 mm, Bregma -2.46 mm. vPmN ventral premammilary nucleus. (TIF) [file pone.0016883.s006.tif]

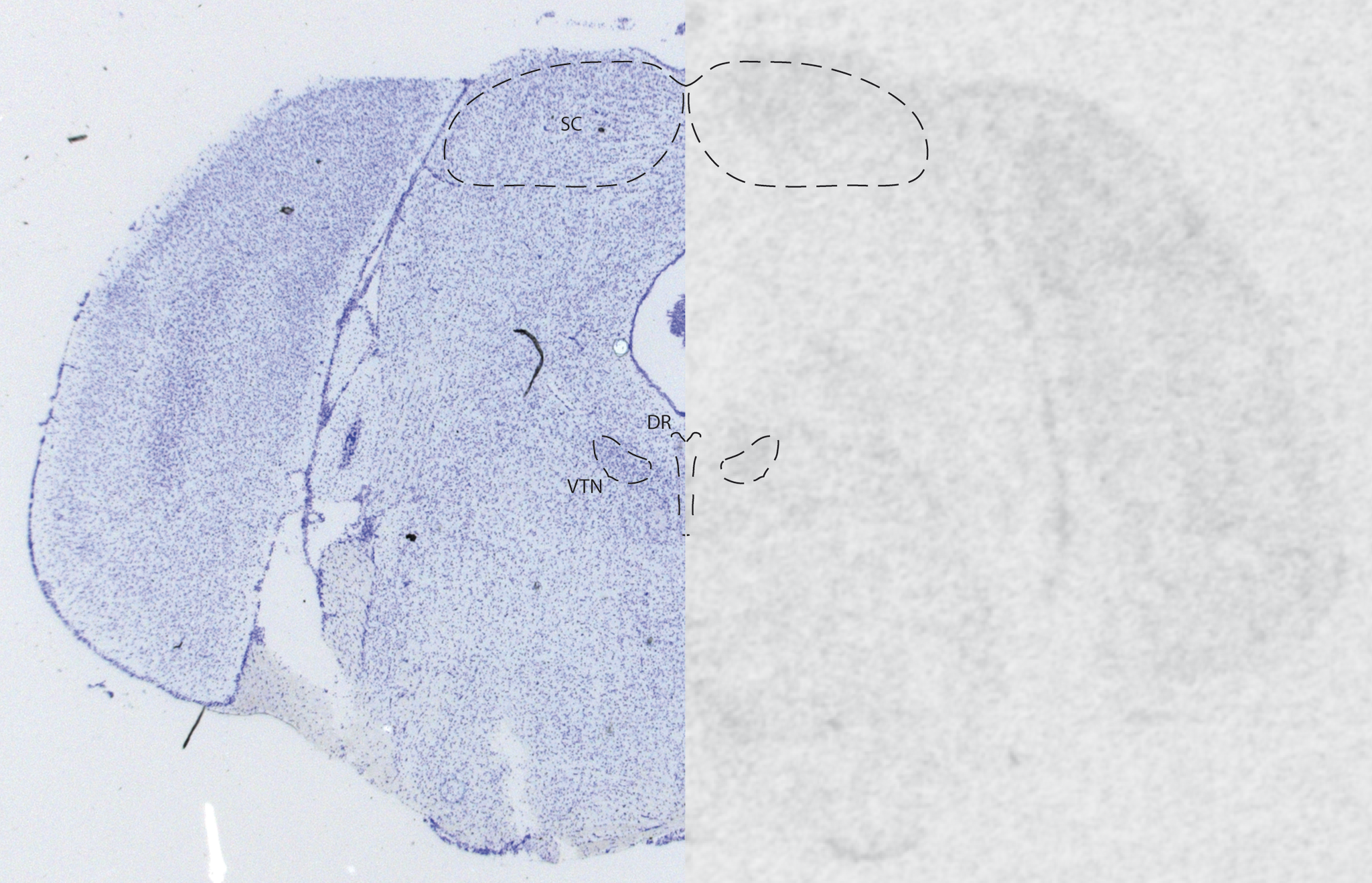

Supplement: Figure S7 — Measured regions for the mapping. Coordinates: Interaural -0.36 mm, Bregma -4.16 mm. SC superior colliculus, DR dorsal raphe nucleus, VTN ventral tegmental nucleus. (TIF) [file pone.0016883.s007.tif]

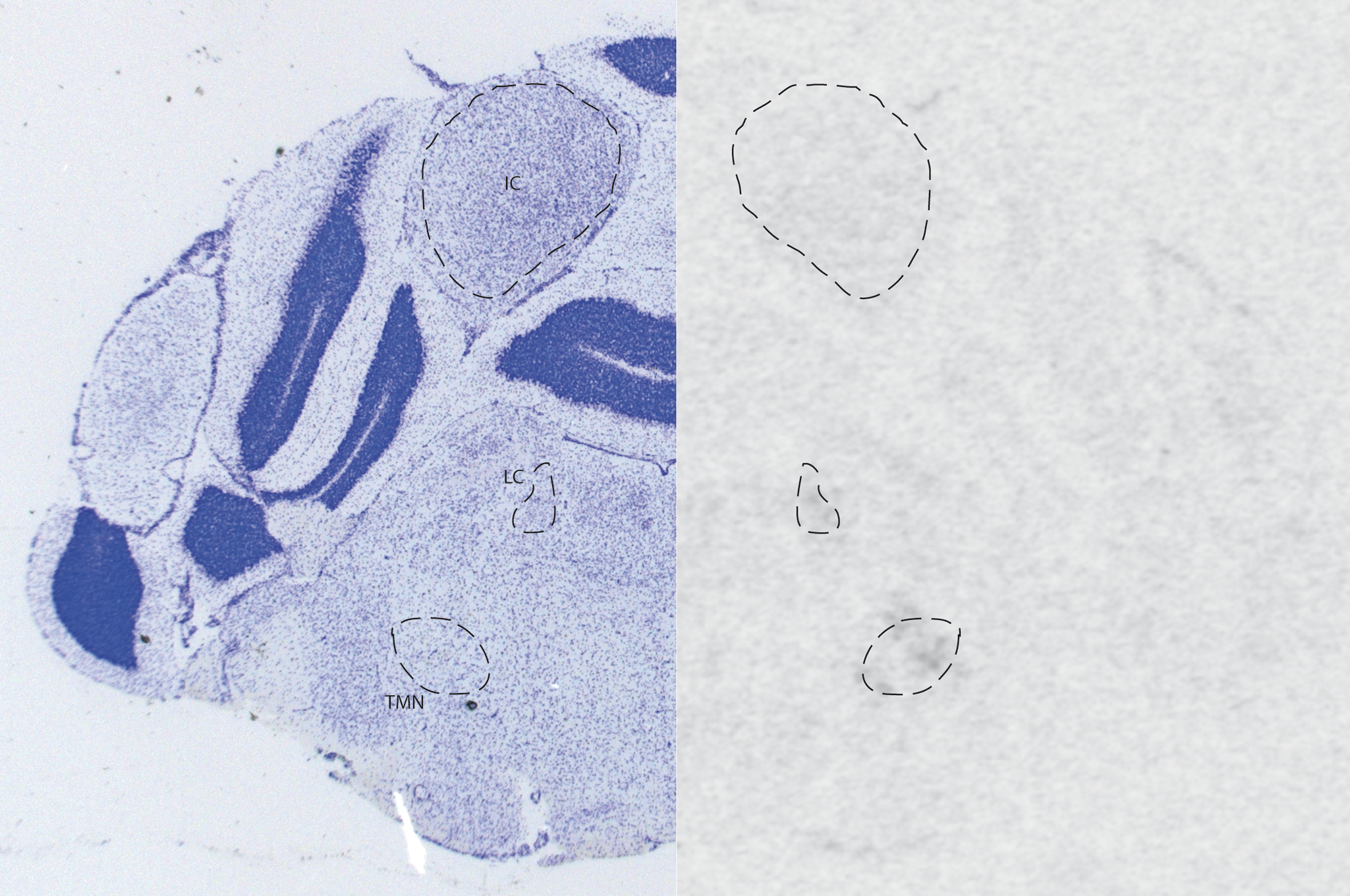

Supplement: Figure S8 — Measured regions for the mapping. Coordinates: Interaural -1.54 mm, Bregma -5.34 mm. LC locus coeruleus, TMN trigeminal motor nucleus, IC inferior colliculus. (TIF) [file pone.0016883.s008.tif]

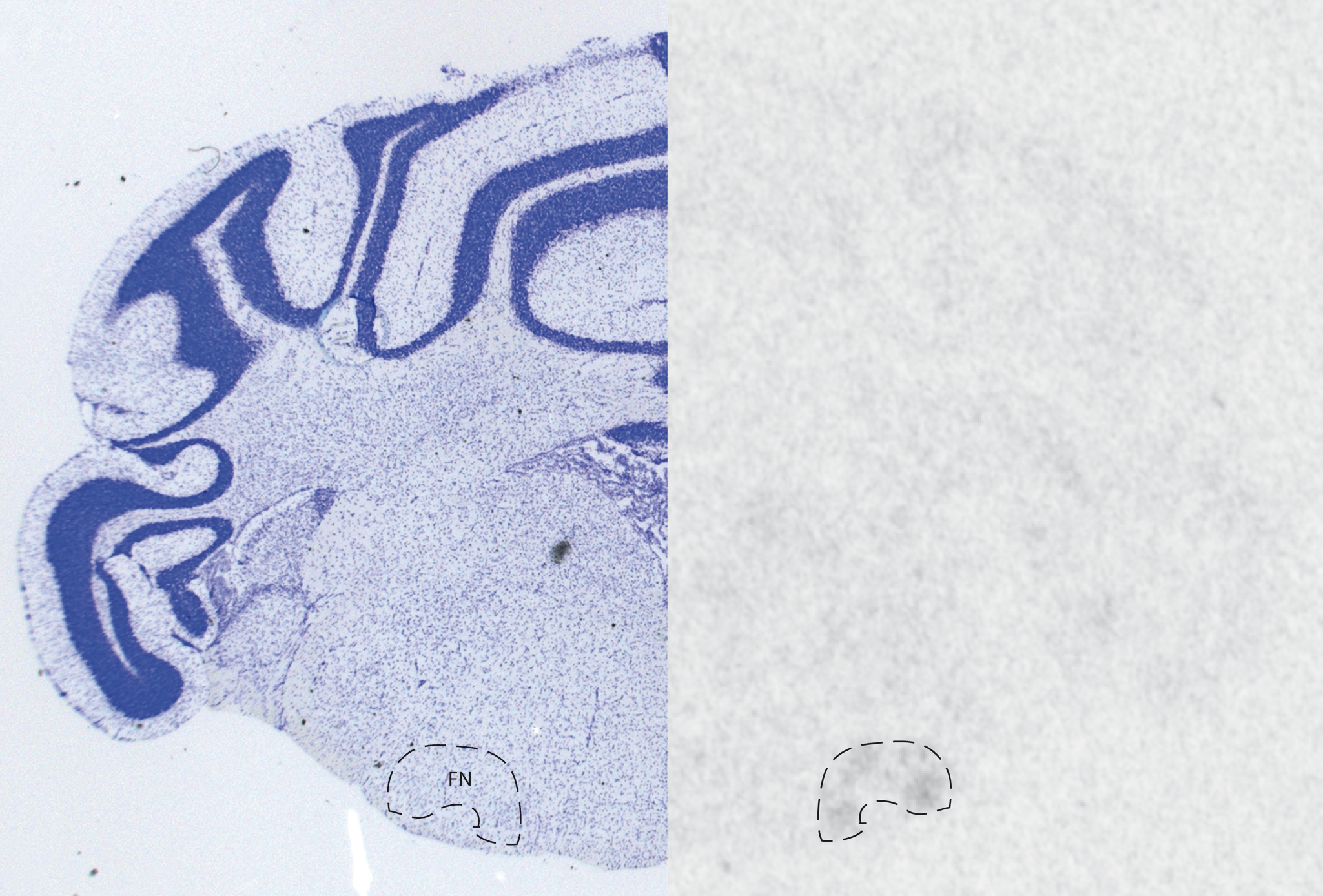

Supplement: Figure S9 — Measured regions for the mapping. Coordinates: Interaural -2.44 mm, Bregma -6.24 mm. FN facial nucleus. (TIF) [file pone.0016883.s009.tif]

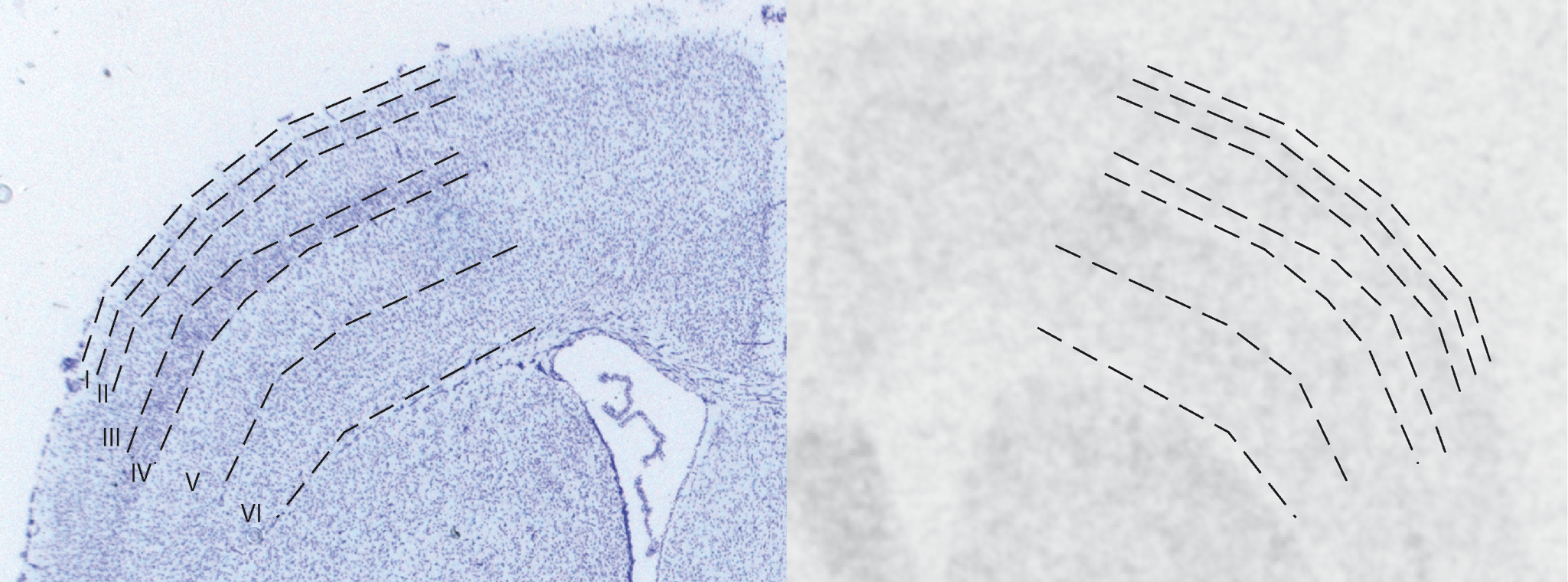

Supplement: Figure S10 — Identification of the cortex layers. Coordinates: Interaural 0.26 mm, Bregma 4.06 mm. Only the cortex is shown. I Layer 1, II Layer 2, III Layer 3, IV Layer 4, V Layer 5, VI Layer 6. (TIF) [file pone.0016883.s010.tif]
